# Enhancing return-to-work in cancer patients, development of an intervention and design of a randomised controlled trial

**DOI:** 10.1186/1471-2407-10-345

**Published:** 2010-07-01

**Authors:** Sietske J Tamminga, Angela GEM de Boer, Jos HAM Verbeek, Taina Taskila, Monique HW Frings-Dresen

**Affiliations:** 1 Coronel Institute of Occupational Health, Academic Medical Center, University of Amsterdam, Amsterdam, the Netherlands; 2 Finisch Institute of Occupational Health Field, Kuopio, Finland; 3 Primary Care Clinical Sciences, University of Birmingham, Birmingham, UK

## Abstract

**Background:**

Compared to healthy controls, cancer patients have a higher risk of unemployment, which has negative social and economic impacts on the patients and on society at large. Therefore, return-to-work of cancer patients needs to be improved by way of an intervention. The objective is to describe the development and content of a work-directed intervention to enhance return-to-work in cancer patients and to explain the study design used for evaluating the effectiveness of the intervention.

**Development and content of the intervention:**

The work-directed intervention has been developed based on a systematic literature review of work-directed interventions for cancer patients, factors reported by cancer survivors as helping or hindering their return-to-work, focus group and interview data for cancer patients, health care professionals, and supervisors, and vocational rehabilitation literature. The work-directed intervention consists of: 1) 4 meetings with a nurse at the treating hospital department to start early vocational rehabilitation, 2) 1 meeting with the participant, occupational physician, and supervisor to make a return-to-work plan, and 3) letters from the treating physician to the occupational physician to enhance communication.

**Study design to evaluate the intervention:**

The treating physician or nurse recruits patients before the start of initial treatment. Patients are eligible when they have a primary diagnosis of cancer, will be treated with curative intent, are employed at the time of diagnosis, are on sick leave, and are between 18 and 60 years old. After the patients have given informed consent and have filled out a baseline questionnaire, they are randomised to either the control group or to the intervention group and receive either care as usual or the work-directed intervention, respectively. Primary outcomes are return-to-work and quality of life. The feasibility of the intervention and direct and indirect costs will be determined. Outcomes will be assessed by a questionnaire at baseline and at 6, 12, 18, and 24 months after baseline.

**Discussion:**

This study will provide information about the effectiveness of a work-directed intervention for cancer patients. The intention is to implement the intervention in normal care if it has been shown effective.

**Trial registration:**

NTR1658

## Background

Since the survival rates of cancer have increased considerably in recent years, the long-term side effects of cancer and cancer-related treatments may impact survivors' capabilities to regain normal lives. This implies that some forms of cancer are becoming chronic diseases entailing both poorer overall quality of life compared to the general population and disabling long-term residual symptoms, such as fatigue, depression, pain, and functional limitations [1-3]. One aspect of regaining a normal life after cancer is returning to work, which is often seen by cancer survivors as an important part of recovery [4-6]. Furthermore, the loss of work of cancer survivors is associated with lower quality of life, lower self-esteem, and worse financial situations [7,8]. Unfortunately, not all cancer survivors return-to-work. A meta-analysis showed that the risk of unemployment is 37% higher for cancer survivors compared to healthy controls [9]; cancer survivors also experience work limitations [10,11]. The employer and society at large are also affected due to absenteeism, disability pensions, and loss of productivity [12]. Furthermore, cancer survivors get little advice from their treating physicians about return-to-work issues, and they experience a lack of guidance from their general practitioners or occupational physicians as well [13,14]. Considering these negative work-related side effects for cancer survivors and the lack of attention toward these problems, there is ample room to improve return-to-work with an appropriate intervention.

A systematic review of the literature [15] concerning work-directed interventions for cancer patients showed that well-developed work-directed interventions are limited and that the methodological quality of these studies is moderate. This indicates that a new intervention needs to be developed and that its effectiveness on return-to-work and quality of life should be determined by a randomised controlled trial. Considering the financial impacts on cancer patients and society at large, the direct and indirect costs should also be taken into account.

Return-to-work is only one aspect of survivor care and it should not be dealt with in isolation. Therefore, we developed the intervention in such a way that it fits the shared-care model for cancer survivor care [16]. This model encompasses a work-directed intervention integrated into cancer care as integrated into the occupational physician or general practitioner's care, establishing active communication between these health care professionals.

### Objective

The objective is to describe the development and content of a work-directed intervention to enhance return-to-work in cancer patients and to explain the study design used for evaluating the effectiveness of the intervention.

## Development of the intervention

The development of the work-directed intervention is based on the following: 1) a systematic review concerning the content of work-directed interventions for cancer patients, 2) factors reported by cancer survivors as helping or hindering their return-to-work, 3) focus group data of cancer survivors and of supervisors regarding return-to-work after cancer, 4) vocational rehabilitation literature, and 5) semi-structured interviews with health care professionals.

First, the systematic review described the content of work-directed interventions for cancer patients on the basis of the two most important prognostic factors for return-to-work that can be directly altered by a work-directed intervention: self-perceived work ability and physical workload [5,17]. Of the 19 included studies, 18 addressed work ability and 6 addressed physical workload. The following interventions to address work ability proved to be suitable: making a return-to-work plan, individual counselling/structural guidance, and cognitive behavioural therapy, and provision of an educational leaflet. The interventions that were shown to be suitable to address physical workload were: workplace accommodations and occupational or vocational training.

Second, we incorporated factors reported by cancer survivors as helping or hindering their return-to-work. These factors were measured in a prospective cohort study of Spelten et al. [18] with two-open questions: "*what or whom helped you the most with regard to work resumption or continuing work?" *and *"what or whom hindered you the most with regard to work resumption and continuing work" *[18]. Cancer survivors answered these open-ended questions at 6, 12, and 18 months after their first day of sick leave. We used the International Classification of Functioning (ICF) as a theoretical framework to categorise the answers into factors underlying work-related disability: body structure and functioning, environment-related factors, and person-related factors [19,20]. The most frequently reported factor that aided in return-to-work in the category of body structure and functioning was general health; the most frequently reported hindering factor was fatigue. With regard to environment-related factors, managers, colleagues, and family were the most frequently reported helping factors and workload a hindering factor. For person-related factors, personality was the most frequently reported helping factor, and distrust in the manager was a hindering factor.

Third, the experiences of cancer survivors and supervisors regarding return-to-work after cancer were assessed in three focus groups [14]. A total of 7 cancer survivors and 6 supervisors participated in the focus groups. The following themes were reported by cancer survivors as being the most important: 1) contact with and support from colleagues and supervisors, 2) the occupational physician's and other physician's advice and expertise, and 3) knowledge of the long-term consequences of cancer, such as fatigue and cognitive problems. The following themes were reported by supervisors as being the most important: 1) their own role and that of the occupational physician, 2) return-to-work aspects such as work accommodations, and 3) communication with all stakeholders. The main findings of these focus groups were that support from colleagues and supervisors aids in return-to-work. Lack of knowledge and advice from occupational physicians and lack of communication between all stakeholders hinders return-to-work [14].

Fourth, because the occupational physician is legalised to advise about the return-to-work of sick-listed employees in the Netherlands, he or she should be part of the intervention. In addition, employer participation is essential for preventing impediments. For instance, for the implementation of workplace accommodations, it is important that the employee, employer, and occupational physician agree. Furthermore, the intervention should be carried out close to the workplace and in collaboration with all stakeholders [21].

Fifth, semi-structured interviews with a radiotherapist and four oncology nurses from four different departments in the Netherlands were held to determine the best way to integrate the intervention into usual cancer care. The health care professionals stated that the intervention should be integrated into psycho-oncological care. This seemed to be feasible based on the results of our systematic review [15].  The interviewees' nurses stated that they lack the knowledge to provide this kind of care but that a training course would help to provide this knowledge. Furthermore, the interviewed nurses and radiotherapist believed that the intervention should be carried out individually because of variations in treatment, survival, and importance of work. In addition, the different factors reported by cancer survivors as helping or hindering their return-to-work also confirm the need to provide individual interventions. The interviewees stated that the psycho-oncological care of cancer patients varies enormously between hospital departments with respect to content, length, duration, extensiveness, and provider. These differences also hold for return-to-work, which is not addressed structurally by any of the departments. However, the interviewees believed that cancer patients need structural guidance in their return-to-work process. To achieve this guidance, they advised that meetings need to be scheduled and that communication with the occupational physician should be improved.

## Content of the intervention

Incorporation of the findings described above resulted in a work-directed intervention that encompasses: 1) 4 meetings of 15 minutes each will be held at the hospital as part of the normal consulting hour to start early vocational rehabilitation. These meetings will be carried out as part of normal psycho-oncological care by an oncology nurse, social worker or nurse practitioner. In this article, we will refer to these people as the nurse. Since usual care differs between hospital departments, the organisation of these meetings may vary slightly, 2) one meeting with the participant, the occupational physician, and the supervisor will be held to make a return-to-work plan, and 3) three letters will be send to the occupational physician to enhance communication; two will be from the treating physician and one from the nurse.

### 1) Meetings at the hospital

The aim of the first meeting is to make a list of potential problems concerning return-to-work and to plan the intervention that best suits the individual participant. The first meeting will take place a few weeks after diagnosis, and the nurse will begin by taking a short work-anamnesis. Thereafter, the nurse will give guidance regarding the best way to inform colleagues and supervisors about the participant's illness and to keep them informed during treatment/aftercare. Furthermore, the participant will receive an informational leaflet about cancer [22]. An educational leaflet that consists of 10 steps of advice [23] will be given to the participant and will be used as a guideline for the intervention. Scheduling of the second meeting will depend on the diagnosis, treatment, and preference of the participant; it will be scheduled for a maximum of 10 months after the first meeting. The aim is to schedule this meeting two months before the participant is expected to return-to-work. This would largely occur at the end of medical treatment, because most cancer patients return-to-work thereafter [18]. However, if a participant wants to return-to-work during treatment, the second meeting will be scheduled two months before the return-to-work is expected.

The second meeting starts with a recapitulation of the topics discussed at the first meeting. Then, barriers to return-to-work will be identified, and actions to remove these barriers will be discussed. This information will be sent to the occupational physician. The identification of barriers to return-to-work and actions to remove these barriers are based on the work-anamnesis, physical, and psychosocial restrictions, coping, individual importance of work, participant preference regarding the return-to-work, and the most important prognostic factors for returning to work, such as, older age, lower education, and blue collar work.

The aim of the third and fourth meetings is to evaluate the process of return-to-work. The third meeting will be scheduled for a maximum of 2 months after the second meeting because during the actual return-to-work, different problems might arise. The barriers to return-to-work and actions to remove these barriers that were discussed in the second meeting will be evaluated. If a return-to-work plan has been made, it will be discussed. If necessary, the return-to-work plan will be altered, and extra information or advice will be provided. Possible medical or psychosocial problems will be discussed, and the patient can be referred to another professional (e.g. a psychologist) if needed. There are two options for the fourth meeting depending on whether the participant has returned to work or not. If a participant has returned to work, the fourth meeting will be scheduled approximately 1 month after the return-to-work, and advice regarding continued employment will be provided. If the participant has not returned to work, the fourth meeting will take place a maximum of 14 months after baseline, and the contents of the third meeting will be discussed.

### 2) Meeting with participant's occupational physician and supervisor

The occupational physicians will be asked to schedule a meeting with the participant, the participant's supervisor, and with himself/herself to make a return-to-work plan. Medical information from the hospital regarding disease, treatment, and long-term side effects as the identified barriers to return-to-work and actions to remove these barriers that were discussed with the nurse can be used as a basis for this discussion. This meeting should be scheduled between the second and third meeting with the nurse, which would be between the second and tenth month after the first day of sick leave. The researcher will request this meeting; the request will be attached to the first letter from the treating physician to the occupational physician. A return-to-work plan will contain the following information: first day of work resumption, the number of hours the participant will work, the task(s) he/she is going to perform, and with which steps patients will increase working hours, working days and/or will do additional or different tasks.

### 3) Enhancing communication between treating physician and occupational physician

In the Netherlands, patients must give their consent to allow medical information to be sent from a treating physician to an occupational physician. A nurse will ask for this consent from each participant during the first meeting. If the participant gives consent, a copy of a letter from their treating physician to their primary care physician (containing general medical information such as diagnosis, prognosis, treatment plan, and outcome treatment) will be sent to their occupational physician. General information about the study (including the educational leaflet) will be attached to this letter. The identified barriers to return-to-work and actions to remove these barriers that were discussed with a nurse will be sent by a nurse to an occupational physician.

## Study design to evaluate the intervention

For the description of the design of our evaluation study, we used the items of the CONSORT statement for improving the quality of reporting randomised trials [24].

### Organisation study

The study is designed as a randomised controlled trial with a follow-up of 2 years. Cancer patients are asked to participate at the hospital. The medical ethics committee of the institution, the Academic Medical Center, approved the study, and the local medical ethics committees of various participating hospitals advised positively about the local feasibility of the study. Patients will sign informed consent forms before participating in the study.

### Recruitment of study population

The treating physician or nurse will inform the cancer patients about the study when they visit the hospital to discuss their treatment plans. The treating physician will check each patient's eligibility by assessing the inclusion and exclusion criteria and provide written information about the study. Then, the researcher will contact the patient by telephone to provide additional information and schedule a meeting with the patient following a visit to the hospital or at the patient's home. After the patient has signed informed consent and filled out a baseline questionnaire, he or she will be randomised to either the control group or the intervention group.

### Participants

Patients are eligible to participate when they have a primary diagnosis of cancer, will be treated with curative intent, are employed at the time of diagnosis, are sick-listed, and are between 18 and 60 years old. Treatment with curative intent was defined as a 1 year survival rate of approximately 80%. The exclusion criteria are: 1) patients who are not able to speak, read or write Dutch sufficiently well, 2) patients who have a severe mental disorder or other severe co-morbidity, 3) Primary diagnosis of cancer has been made more than two months ago, 4) patients who will receive primary treatment at another hospital, and 4) patients who have a primary diagnosis of testicular cancer, non-melanoma skin cancer or melanoma skin cancer. This last exclusion criterion has been selected because, on average, these cancer patients do not experience significant problems with return-to-work; therefore, the intervention would not be useful for the majority of this patient group [5,25].

### Randomisation, blinding and treatment allocation

The researcher will carry out randomisation using the computerised randomisation program ALEA [26]. Allocation of each patient is definite in such a way that allocation concealment is not possible. Because, patients differ significantly between the participating hospital departments in diagnosis and demographic factors, and because these aspects, as well as age, are important prognostic factors for return-to-work, [18] randomisation is stratified by the treating hospital department and age to prevent bias due to unequal randomisation. The cut-off age for stratification is 50. To equalise group sizes, minimisation is applied. If a treating hospital department has more than one nurse, participants in the intervention group will only be seen by nurses who carry out the intervention and vice versa. Participants, nurses, and researchers are not blinded to group assignment. Because all follow-up questionnaires will be filled out at home, no direct influence by the researchers is likely to occur.

### Sample size

The calculation of sample size is based on two earlier studies regarding return-to-work in cancer patients. Based on the study by Spelten et al. [18] with consecutive cancer patients, the expectation is that 18 months after diagnosis 36% of patients will not have returned to work [18] after care as usual in the control group. With regard to the intervention group, the expectation is that 19% of the patients will not have returned to work 18 months after diagnosis [23], based on the study by Nieuwenhuijsen et al. [23]. Due to the inclusion and exclusion criteria, a percentage of patients with relatively less severe return-to-work problems will not be included in this study and this may lead to less favourable return-to-work rates. However, our intervention is more comprehensive than in the study by Nieuwenhuijsen et al. [23] and thus the expectation is that the percentages of patients that will not have returned to work will be the same as in the study by Nieuwenhuijsen et al. [23]. This indicates an Odds Ratio of 0.41 of the intervention versus usual care for higher percentages of patients that are not returned to work, which is the same as an Odds Ratio of 2.4 of the intervention versus care as usual for higher return-to-work rates. Since, the incidence of outcome is more than 10%, the Odds Ratio overestimates the magnitude of the association and therefore, we calculated the Relative Risk based on the Odds Ratio [27]. This indicates a Relative Risk of not returning to work of 0.53 of the intervention versus usual care. Based on the PS Power and Sample size Program, with a power of 80% and two-sided significance level of p < 0.05, the sample size should be 109 patients in every arm, for a total of 216 patients [28]. Assuming that 20% of the initial patients will be lost to follow-up during the course of the study, 270 patients must be recruited to gather 246 patients at 24 months. To account for at least 10% missing data at baseline, 300 patients are intended to be included. Furthermore, a sample size of 300 will have sufficient power to be able to control for the prognostic factors in a Cox regression analysis since we assume that 5 to 10 variables will be included in the final model [17,18] and a sample size of 10 per included factor in a Cox regression analysis is considered sufficient [29].

### Contamination

In departments with more than one nurse, nurses who counsel participants in the intervention group will not counsel participants in the control group, and vice versa. Nurses who counsel participants in the intervention group are asked not to discuss the content of the intervention with nurses who counsel participants in the control group. In departments with only one nurse, this separation is not possible. However, since all nurses in the intervention group will need to extend their meeting for the intervention, and since they all need to fill out a form during each meeting, contamination between groups will be diminished. It is unlikely that employees of the same company will participate in the study; therefore, it is unlikely that participants in the control group will receive detailed information about the content of the intervention. It is possible that participants in the intervention group will discuss the content of the intervention with those in the control group at the hospital in the waiting room or during chemotherapy treatment. However, we do not consider this possibility a serious risk of contamination.

Participants will be able to use any co-intervention they wish. Since it is likely that other vocational rehabilitation programs will have a significant effect on return-to-work, these co-interventions will be monitored by asking the participants at the end of the intervention if they have participated in any other vocational rehabilitation programs. Because the effect of other rehabilitation programs on return-to-work is unknown, these co-interventions will not be assessed.

### Usual care in the Netherlands

Employees who are diagnosed with cancer should be guided according to the blueprint of evidence-based guidelines of the Dutch Association of Occupational Physicians (NVAB) [30]. Furthermore, sick leave is covered in the Netherlands by the Improved Gatekeepers Act, which is in force during the first 2 years of sick leave. The act states that a sick-listed employee cannot be discharged and receives at least 70% of his/her wage. After 2 years of sickness, the sick-listed employee will be assessed for disability pension. The Improved Gatekeepers Act states that employers and sick-listed employees are responsible for work resumption, which means that both parties can be sanctioned. Furthermore, all sick-listed employees should have an occupational physician who should legally advise them about return-to-work.

### Training nurses to carry out the intervention

The nurses who carry out the intervention will participate in a half-day training course. This training course consists of education about the rights and obligations of sick-listed employees according to the Improved Gatekeepers Act and education about the return-to-work problems of cancer patients. For instance, education about the return-to-work rates of cancer patients, factors that can have an impact on the return-to-work outcomes (e.g. diagnosis, type of treatment, and work ability), and the role of colleagues, supervisors, and occupational physicians. Furthermore, extensive practice is given at each meeting by means of role-playing and discussions between trainer and trainees. The training course will be given by a trained psychologist and a researcher. For evaluation, all nurses are asked to give their opinions of the training course by filling out an anonymous questionnaire at the end of the course.

### Outcomes

The primary outcome parameters are return-to-work and quality of life. The secondary outcome parameters are work ability, work limitations, study feasibility, and direct/indirect costs of the intervention. The primary and secondary outcomes will be assessed at baseline and at 6, 12, 18, and 24 months after baseline. Participants will fill out the baseline questionnaires directly after signing the informed consent forms. The other questionnaires will be mailed with an enclosed free return envelope. It will take participants approximately 30 minutes to complete each questionnaire. The questionnaire has been pilot-tested with healthy subjects and cancer patients.

#### Effect evaluation

The study's effectiveness will be determined on the basis of the primary outcomes of return-to-work and quality of life and the secondary outcomes of work ability and work limitations, assessed at the long-term follow-up at 12 months and at the very long-term follow-up at 24 months. Prognostic factors will be taken into account in the effect analysis only if there are imbalances between the intervention group and the control group. Effectiveness will be inferred if participants in the intervention group will have a significantly shorter time to return-to-work than participants in the control group and if at the same time their quality of life does not significantly deteriorate. Return-to-work is measured both as the number of calendar days between the first day of sick leave and the first day at work (either part-time or full-time) sustained for at least 4 weeks and as the rate of return-to-work at follow-up. The first day of sick leave, the number of calendar days until return-to-work, and the return-to-work rates will be based on patient self-reporting on questionnaires [23]. Quality of life will be assessed with the SF-36 [31]. All subscales of the SF-36 will be taken into account (physical functioning, role limitations due to physical health problems, bodily pain, general health perceptions, vitality, social functioning, role limitations due to emotional problems, and general mental health). The SF-36 has been validated in a sample of cancer patients, and normative values have been determined [31]. Work ability will be assessed with the first 3 questions of the Work Ability Index (WAI) [32]. These questions concerns the evaluation of current work ability compared to their life time best and current physical and mental work ability with respect to their job demands. Acceptable measures for reliability and validity have been determined [33,34]. Work limitations will be assessed on the basis of the Work Limitation Questionnaire, where work limitations are defined as being inversely related to productivity [35]. This questionnaire consists of four different subscales: work scheduling, physical demands, mental demands/social demands, and output demands. The English version of the Work Limitation Questionnaire has been proven valid and reliable in populations of several chronic diseases [35] and cancer survivors [10,11,36]. The validity and reliability assessments of the Dutch translation of the Work Limitation Questionnaire are currently underway for healthy controls and cancer patients.

#### Process evaluation

Process evaluation is divided into the following parameters: 1) feasibility of the procedure, 2) satisfaction with the intervention, 3) participant compliance with the intervention, 4) nurse adherence to the protocol, and 5) evaluation of usual care. First, feasibility of the procedure will be assessed by a researcher on the basis of a checklist at the end of the study. Second, nurse and participant satisfaction with the intervention will be assessed by a questionnaire. Third, patient compliance with the intervention will be assessed by a questionnaire. Fourth, nurse adherence to the protocol will be assessed by a researcher on the basis of reports from the nurse. Fifth, evaluation of usual care will be assessed on the basis of a questionnaire and includes organisational factors such as received support from the organisation and the occupational physician.

#### Economic evaluation

For the economic evaluation, the work-related costs to society, the individual cancer patient, and the employer will be taken into account, since everyone incurs costs when an individual cancer patient does not return-to-work [37]. In this way, the costs and benefits will be calculated independently of those who bear these costs and those who receive the benefits. For the intervention group, direct costs such as the costs to carry out the intervention, and indirect costs, such as absenteeism or work productivity, lost earnings, and work adjustments will be taken into account. For the control group direct costs such as the costs to carry out care as usual, and indirect costs, such as absenteeism or work productivity, lost earnings, and work adjustments will be taken into account. The direct costs will be determined by means of the average nurse wage and the amount of time spent on each participant. The nurse will record the duration of each meeting at its completion. Indirect costs will be obtained by a questionnaire. Absenteeism will be determined by means of days on sick leave and on income and work productivity by the Work Limitation Questionnaire and on income [35]. Furthermore, lost earnings will be determined on the basis of the differences between income at baseline and income at follow-up. Work adjustments will be assessed by means of the cost of each work adjustment.

#### Prognostic and descriptive factors

We have taken into account as prognostic factors, all factors that were significant related to time to return-to-work in a prospective cohort study on the impact of cancer-and work-related factors on the return-to-work of cancer patients [17,18]. Prognostic factors will be assessed by questionnaires at all time points except for diagnosis and treatment, which will be retrieved from patient files. The prognostic factors include age, gender, education, diagnosis, cancer treatment, number of working hours according to contract, physical workload as measured by the Questionnaire Perception and Judgement of Work (VBBA) [38], importance of work as measured by a Visual Analogue Scale (VAS-scale), fatigue as measured by the Multidimensional Fatigue Inventory (MFI) [39], depression as measured by the Centre for Epidemiologic Studies for Depression Scale (CES-D) [40], co-morbidity, income, self-efficacy as measured by the general self-efficacy scale (ALCOS) [41], and global quality of life as measured on a VAS-scale. The descriptive factors include the number of days between the first day of sick leave and inclusion in the study, marital status, ethnicity [42], time since diagnosis, breadwinner status, position at work, shift work, years in current position, years of paid employment, and company size. These descriptive factors will be measured only at baseline.

### Statistical analysis

#### Effectiveness

All analyses, which will be performed to distinguish differences between the control group and intervention group, will be performed according to the intention-to-treat principle. All baseline data and data regarding primary and secondary outcomes will be presented using descriptive statistics. The number of days until participants' return-to-work will be analysed using the Kaplan-Meier survival method, and differences between groups will be tested with a log rank test. If necessary, the differences between the control group and the intervention group will be adjusted with a Cox regression analysis for confounders such as diagnosis and for the prognostic parameters. Longitudinal multilevel analysis will be used to examine differences between the control group and the intervention group with regard to improvement of the primary outcome of quality of life and the secondary outcomes of work ability and work limitations.

#### Economic evaluation

The economic evaluation will be performed according to the intention-to-treat principle. The direct and indirect costs will be summed for each participant. Mean differences in direct, indirect, and total costs will be calculated between the control group and the intervention group using bootstrapping. Incremental cost-effectiveness ratios will be calculated by assessing the ratio between the differences in costs between the groups to the differences in return-to-work rates between the groups. These ratios will be displaced in a cost-effectiveness plane, and acceptability curves will be presented.

## Discussion

There is a lack of effective work-directed interventions specifically tailored to cancer patients. The aim of this study is to develop an intervention that will be carried out as a randomised controlled trial.

As a starting point for developing a new work-directed intervention, the shared care model, adjusted for the vocational rehabilitation setting, has been used. The work-directed intervention involves: 1) 4 meetings with a nurse at the treating hospital department to start early vocational rehabilitation, 2) 1 meeting with the participant, occupational physician, and supervisor to make a return-to-work plan, and 3) letters from a treating physician to an occupational physician to enhance communication. The aim of the work-directed intervention is to improve cancer patients' care and to enhance their return-to-work and quality of life.

### Methodological considerations of the development and content of the intervention

#### Developmental considerations

In the literature, methods such as intervention mapping are often used as tools for developing new interventions. In this study, no such tools have been used; however, all of the important ingredients to develop an intervention have been employed, including the use of a model, literature, and expert knowledge. By putting this intervention into practice, the quality of vocational rehabilitation might improve due to the improved medical knowledge of an occupational physician about each patient and improved continuity of care [43].

#### Content considerations

Because we used a cancer care model and interviews with health care professionals we think that we have taken practical considerations into account that will improve the intervention implementation. This resulted for example in an extra burden on participants and health care professionals that is in our view quite reasonable.

### Methodological considerations of the study design

We used the items of the CONSORT statement for improving the quality of reporting randomised trials as guidance for the study protocol [24]. One drawback of our study design is that in departments with one nurse, the nurse might also give better guidance to participants in the control group leading to contamination. One way to prevent this kind of contamination is to randomise on the hospital department level, so-called cluster randomisation. In our case this had the following disadvantages. Cluster randomisation on the department level will irrevocably lead to baseline differences in patient characteristics, such as diagnosis, treatment, and age because departments deal with specific cancer patients. These baseline characteristics are the most important prognostic factors for return-to-work [17,18] and differ significantly between hospital departments in the Netherlands. Although it is possible to correct for these baseline differences afterward, bias will always be apparent. Further, nurses might invite a specific group of patients to participate in the study since they know in which group each patient will come [44]. At last, psycho-oncology care differs between hospital departments; thus, the outcome might be biased by these differences in usual care. The drawback of our approach is of course contamination. To reduce the likelihood of contamination, nurses must fill out a process form for each patient in each meeting.

We choose not to perform a comprehensive cost effectiveness evaluation in which all direct and indirect costs, such as medical consumption are taken into account because the expectation is that the intervention has no effect on these costs. Therefore, the expectation is that these medical costs are equally divided between groups and therefore do not contribute to the cost analysis. In addition, the aim of our work-directed intervention is to improve participants' return-to-work and work productivity and to reduce absenteeism and lost earnings. Therefore, we choose to perform the economic evaluation on the basis of these work-related costs.

This study will provide information about the effectiveness of our work-directed intervention on return-to-work and quality of life. The results will be available in 2011. Furthermore, the intention is to implement the intervention in normal care if it has been shown effective.

## Competing interests

The authors declare that they have no competing interests.

## Authors' contributions

ST is responsible for data collection and drafted the manuscript. AdB, JV, and MF obtained funding for this study. All authors conceived and designed the study, and read and corrected draft versions of the manuscript, and approved the final manuscript.

## Pre-publication history

The pre-publication history for this paper can be accessed here:

http://www.biomedcentral.com/1471-2407/10/345/prepub
